# Analysis of Risk Factors and Circumferential Distribution of Exposed Cardiac Glands

**DOI:** 10.1002/jgh3.70241

**Published:** 2025-10-29

**Authors:** Mingyang Fan, Haiyang Hua, Jingyi Yin, Chunrou Long, Yuan Li, Jianhui Li, Xin Hao

**Affiliations:** ^1^ Chengde Medical College Chengde China; ^2^ Department of Gastroenterology Chengde Central Hospital Chengde China

**Keywords:** circumferential distribution, exposed cardiac glands, risk factors

## Abstract

**Objective:**

To investigate the risk factors associated with exposed cardiac glands and their circumferential distribution in the esophagus and to establish a theoretical foundation for the occurrence and progression of these glands.

**Methods:**

Prospectively enrolled patients who underwent gastroscopy in our hospital from December 2023 to March 2024. Patients who met the inclusion and exclusion criteria and were found to have exposed cardiac glands during gastroscopy were included in the exposed cardiac glands group, while those who met the inclusion and exclusion criteria without exposed cardiac glands during endoscopy were included in the control group. The risk factors for exposed cardiac glands were obtained through statistical analysis by comparing various factors between the two groups. The circumferential distribution of exposed cardiac glands in the esophagus was studied by means of a radar map.

**Results:**

Waist circumference, drinking, irritating food, right side sleeping position, calcium antagonists, *Helicobacter pylori* infection, and gastroesophageal reflux disease were the risk factors for exposed cardiac glands. The exposed cardiac glands usually occur on the lesser curved side and posterior wall of the distal esophagus.

**Conclusion:**

There is a correlation between exposed cardiac glands and gastroesophageal reflux disease, and the prone site of exposed cardiac glands is roughly consistent with the prone site of reflux. Affected by reflux, exposed cardiac glands may coalesce and progress. Waist circumference, drinking, irritating food, right side sleeping position, calcium antagonists, and *Helicobacter pylori* infection were independent risk factors for exposed cardiac glands.

## 1 Introduction

The cardiac glands of the esophagus are located in the lamina propria of the mucosa approximately 1 cm proximal to the esophagogastric junction (EGJ) [1], and are characterized by columnar epithelium. When the esophageal cardiac glands are directly exposed to the surface of the esophageal mucosa from the mucous lamina propria, they are called the exposed cardiac glands. Under endoscopy, the exposed cardiac glands appear as single or multiple columnar epithelium islands about 1 cm from the proximal end of the EGJ [2]. Only a few foreign studies have shown that reflux may lead to the emergence of exposed cardiac glands, and under the continuous stimulation of reflux, exposed cardiac glands may coalesce and progress into the transitional epithelium of Barrett mucosa [2, 3]. The metaplastic types of Barrett's esophagus (BE) include cardiac epithelial metaplasia, glandular cardiac epithelial metaplasia, and specialized intestinal metaplasia. Among these, BE with intestinal epithelial metaplasia carries a higher risk of carcinogenesis. In contrast, exposed cardiac glands refer to normal cardiac glands located above the EGJ that become exposed on the surface of the distal esophagus due to anatomical variations or gastroesophageal reflux. These glands are histologically normal gastric cardiac glands that secrete mucus to protect the mucosa, lack intestinal metaplasia, and do not require aggressive treatment. Therefore, exposed cardiac glands are primarily distinguished from specialized intestinal metaplasia‐associated BE. Because there are relatively few studies on this subject at home and abroad, this paper discusses the risk factors for the development of exposed cardiac glands and their circumferential distribution in the distal esophagus, aiming to provide some theoretical support for the development of exposed cardiac glands. Since BE in China is defined as the dentate line moving up ≥ 1 cm relative to the EGJ [4], in order to distinguish it, the exposed cardiac glands were defined in this study as the single or multiple columnar epithelium islands appearing within 1 cm above the EGJ.

## 2 Object and Method

### 2.1 Object Selection

**FIGURE 1:**
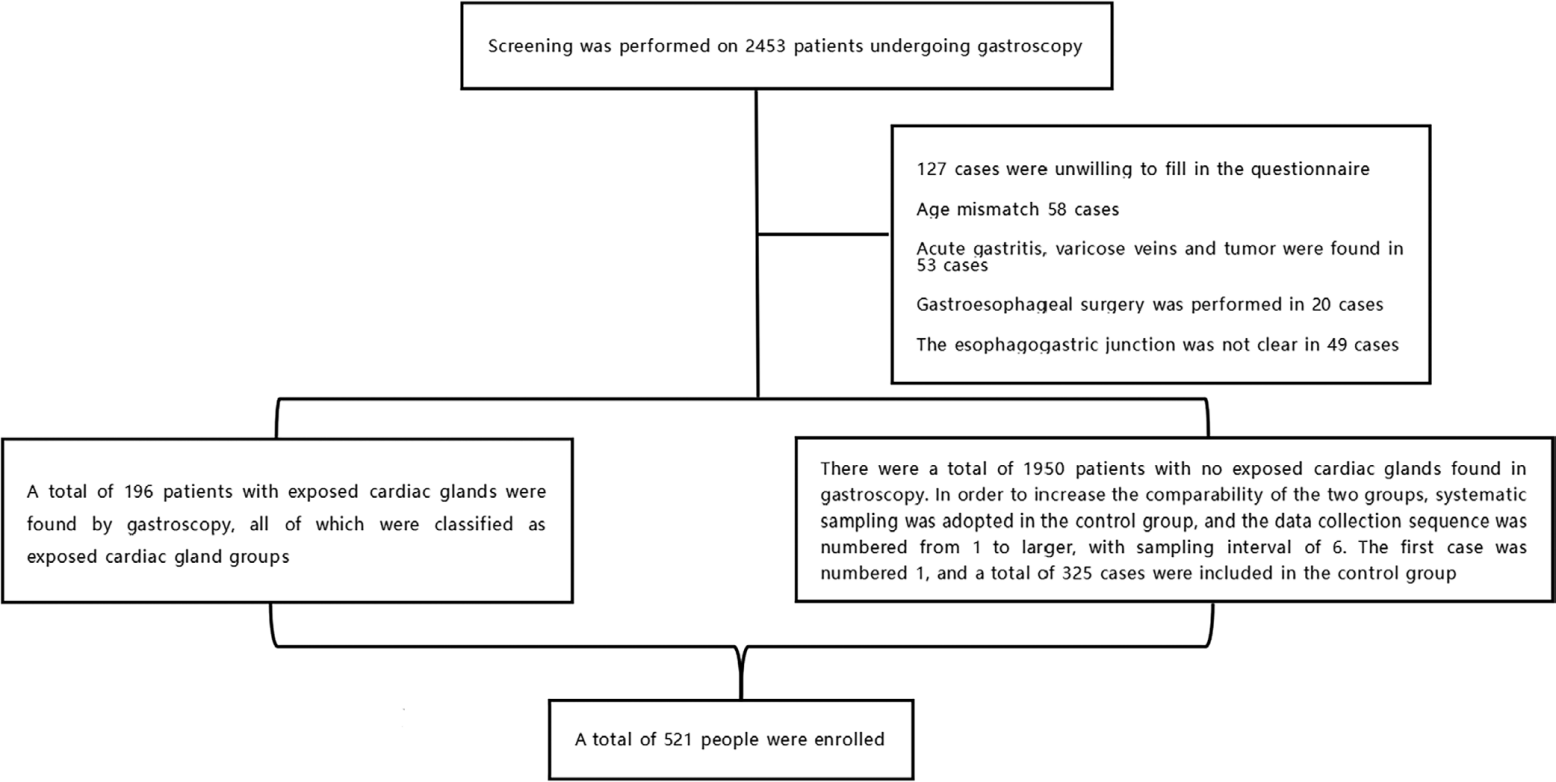
Grouping flowchart.

Patients who underwent gastroscopy at Chengde Central Hospital between December 2023 and March 2024 were prospectively enrolled based on their fulfillment of the predefined inclusion and exclusion criteria. Inclusion criteria: (1) Voluntary acceptance of general demographic characteristics questionnaire, Gastroesophageal reflux disease questionnaire (GerdQ) scale, and gastroscopy, and informed consent signed by patients or their families; (2)Patients or their family members consent to the inclusion of the patient's clinical data in the study; (3) Gastroscopy must be performed by physicians with at least 5 years of experience in performing diagnostic endoscopic procedures. Exclusion criteria: (1) Age < 18 years or > 75 years; (2) Pregnant or lactating women; (3) Patients with acute inflammation of the digestive tract, esophagogastric varices, gastroesophageal tumors, etc.; (4) Patients who have previously undergone gastroesophageal surgery; (5) Patients who cannot cooperate with gastroscopy, such as patients with shock, mental disorders, and consciousness disorders; (6) Patients with severe cardiopulmonary diseases such as acute myocardial infarction and acute attack of chronic obstructive pulmonary disease. Those who have been found with exposed cardiac glands in endoscopy and met the above inclusion and exclusion criteria were included in the exposed cardiac glands group. Those without exposed cardiac glands in endoscopy during the same period but who met the above inclusion and exclusion criteria were included in the control group. Finally, 196 patients were included in the exposed cardiac glands group and 325 in the control group (see Figure 1).

## 3 Inspection Method

### 3.1 Preparation Before Inspection

The patients signed informed consent for gastroscopy before gastroscopy and filled out the general demographic characteristics questionnaire and GerdQ scale by themselves. Fasting for 12 h and water prohibition for 4 h were required before gastroscopy, and 10 mL of the pharyngeal anesthetic lidocaine glue was taken 15–30 min before gastroscopy.

### 3.2 General Demographic Characteristics Questionnaire

This includes gender, age, height, weight, waist circumference, lifestyle habits (e.g., smoking, drinking, tea, coffee, and preference for irriting food), habitual sleeping position (supine, left side, right side, none specified), and long‐term medication history (e.g., calcium antagonists, benzodiazepines, non‐steroidal anti‐inflammatory drugs).

### 3.3 GERD‐Q Scale

Patients recalled the number of days of reflux, heartburn, sleep disturbance, and use of over‐the‐counter drugs in the past 1 week, and rated them on a scale of 0, 1–2, 3–4, and 5–7 days.

### 3.4 Check Steps

During the examination, all patients were placed in the left side lying, and detailed observation was made on whether there were exposed cardiac glands within 1 cm above the EGJ. If there were exposed cardiac glands, we would record in detail the number, size, the distance to the EGJ, and location in the distal esophagus. The following conditions were recorded in detail, including the grade of gastroesophageal flap valve in patients, presence of ectopic esophageal and gastric mucosa, gastrointestinal ulcer, bile reflux, mucosal atrophy or intestinalization, *Helicobacter pylori* infection (Hp) and gastroesophageal reflux disease (GERD). In addition, the two authors (Mingyang Fan, Jingyi Yin) reviewed the endoscopic films to clarify the accuracy of endoscopic information.

## 4 Relevant Definitions and Diagnostic Criteria

### 4.1 Exposed Cardiac Glands

Single or multiple columnar epithelium islands within 1 cm from the proximal end of the EGJ under endoscopy.

### 4.2 General Demographic Characteristics

Smoking is defined as smoking at least 1 cigarette per day for 6 consecutive or cumulative months [5]; drinking is defined as drinking alcohol at least once a week during the past year [6]; tea and coffee consumption is defined as drinking at least once a week in the past year; preference for irritating food is defined as the consumption of spicy or greasy foods at least 3 days a week for 6 consecutive or cumulative months [5]. Habitual sleeping position is defined as the use of one sleeping position at least 4 days a week for 6 consecutive or cumulative months; long‐term medication history is defined as continuous or cumulative medication use for at least 3 months.

### 4.3 Grade of Gastroesophageal Flap Valve

Hill grading was used, in which grade I had obvious tissue folds and tightly wrapped the endoscope along the small curved side. In grade II, tissue folds are less obvious than in grade I, opening and closing rapidly with respiratory movement. In grade III, tissue folds are almost absent and cannot tightly wrap the endoscope, and hiatal hernia may be present in some patients. Grade IV is the complete disappearance of the tissue fold; the gastroesophageal area remains open, squamous epithelium can be seen in the inversion position, and hiatal hernia is often present [7].

### 4.4 Mucosal Atrophy or Intestinalization

Confirmed by final pathological examination.

### 4.5 Hp Infection

Confirmed by 13C breath test or pathological HP test.

### 4.6 GERD

Including Reflux esophagitis (RE) and Non‐erosive reflux disease (NERD). If mucosal damage and erosion were found in the distal part of the esophagus, RE was diagnosed, and the location of mucosal injury of RE was recorded. If the RE diagnosis is not established but there are typical acid reflux and heartburn symptoms and GerdQ scale ≥ 8 points, the diagnosis is NERD [8].

### 4.7 Statistical Method

Data analysis was performed using SPSS software, version 26.0. Quantitative data was represented by median, and qualitative data was represented by case number and percentage. Univariate and multivariate logistic regression were used to analyze the independent risk factors for exposed cardiac glands. A receiver‐operating characteristic (ROC) was established to evaluate the predictive efficacy of exposed cardiac glands on GERD. *p* < 0.05 was considered statistically significant. The basic characteristics of exposed cardiac glands were described, and the proportion of exposed cardiac glands in different directions in the distal esophagus was indicated by radar map.

## 5 Result

### 5.1 Baseline Feature

After screening, a total of 521 patients were enrolled (Figure 1). Among them, 270 cases (51.8%) were male and 251 cases (48.2%) were female. The median age of patients was 55.00 (44.00–63.00) years. The median body mass index (BMI) was 24.47 (22.75–26.23) kg/m^2^. The median waist circumference was 79.00 (72.50–84.50) cm. In terms of living habits, 210 people (40.3%) liked to eat irritating food; relatively few patients took benzodiazepines (7.3%) and non‐steroidal anti‐inflammatory drugs (8.4%) for a long time. In terms of endoscopic factors, 218 patients (41.8%) had GERD; there were relatively few patients with ectopic esophageal and gastric mucosa (5.6%), bile reflux (8.4%) and gastrointestinal ulcer (6.7%). The general demographic characteristics and endoscopic factors are shown in Table 1.

**TABLE 1:** Baseline characteristics of enrolled patients.

Variable	Total (*n* = 521)
Gender (Male/Female)	270/251 (51.8%/48.2%)
Age (year)	55.00 (44.00–63.00)
BMI (kg/m^2^)	24.47 (22.75–26.23)
Waist circumference (cm)	79.00 (72.50–84.50)
Smoking	144 (27.6%)
Drinking	171 (32.8%)
Tea	137 (26.3%)
Coffee	64 (12.3%)
Irritating food	210 (40.3%)
Sleeping position	
Supine	116 (22.3%)
Left side	130 (25.0%)
Right side	115 (22.1%)
None specified	160 (30.7%)
Calcium antagonistss	137 (26.3%)
Benzodiazepines	38 (7.3%)
Non‐steroid anti‐inflammatory drugs	44 (8.4%)
Grade of gastroesophageal flap valve	
Grade I + II	460 (88.3%)
Grade III + IV	61 (11.7%)
Ectopic esophageal and gastric mucosa	29 (5.6%)
Bile reflux	44 (8.4%)
Gastrointestinal ulcer	35 (6.7%)
Mucosal atrophy/intestinalization	187 (35.9%)
Hp infection	100 (19.2%)
GERD	218 (41.8%)

### 5.2 Risk Factors for Exposed Cardiac Glands

Baseline characteristics of both groups were shown in Table 2. Subsequently, univariate and multivariate Logistic regression analyses were used to explore risk factors for exposed cardiac glands. Multivariate Logistic regression analysis showed that waist circumference (OR = 1.078, 95% CI: 1.020–1.138, *p* = 0.008), drinking (OR = 2.376, 95% CI: 1.420–3.976, *p* = 0.001), irritating food (OR = 1.771, 95% CI: 1.100–2.849, *p* = 0.019), right side sleeping position (OR = 3.020, 95% CI: 1.568–5.816, *p* = 0.001), calcium antagonists (OR = 4.512, 95% CI: 2.634–7.728, *p* < 0.001), Hp infection (OR = 3.073, 95% CI: 1.692–5.581, *p* < 0.001) and GERD (OR = 3.067, 95% CI: 1.919–4.902, *p* < 0.001) were significantly associated with exposed cardiac glands (*p* < 0.05). The above factors are all risk factors.

**TABLE 2:** Univariate and multivariate logistic regression analysis of patients in the two groups.

Variable	Exposed cardiac glands group (*n* = 196)	Control group (*n* = 325)	Univariate		Multivariate	
			OR (95% CI)	*p*	OR (95% CI)	*p*
Gender (Male/Female)	106/90 (54.1%/45.9%)	164/161 (50.5%/49.5%)	1.156 (0.811–1.649)	0.423		
Age (year)	54.00 (42.00–62.50)	55.00 (45.00–63.00)	0.995 (0.981–1.010)	0.523		
BMI (kg/m^2^)	25.95 (23.44–27.68)	24.22 (22.60–25.39)	1.245 (1.161–1.336)	< 0.001*		
Waist circumference (cm)	83.00 (75.00–90.00)	78.00 (70.50–82.00)	1.084 (1.059–1.109)	< 0.001*	1.078 (1.020–1.138)	0.008*
Smoking	74 (37.8%)	70 (21.5%)	2.210 (1.494–3.269)	< 0.001*		
Drinking	94 (48.0%)	77 (23.7%)	2.968 (2.031–4.337)	< 0.001*	2.376 (1.420–3.976)	0.001*
Tea	74 (37.8%)	63 (19.4%)	2.523 (1.693–3.759)	< 0.001*		
Coffee	32 (16.3%)	32 (9.8%)	1.787 (1.056–3.023)	0.031*		
Irritating food	97 (49.5%)	113 (34.8%)	1.838 (1.281–2.638)	0.001*	1.771 (1.100–2.849)	0.019*
Sleeping position			1.598 (1.356–1.883)	< 0.001*		
Supine	41a (20.9%)	75a (23.1%)				
Left side	46a (23.5%)	84a (25.8%)				
Right side	70b (35.7%)	45a (13.8%)			3.020 (1.568–5.816)	0.001*
None specified	39b (19.9%)	121a (37.2%)				
Calcium antagonistss	87 (44.4%)	50 (15.4%)	4.390 (2.906–6.632)	< 0.001*	4.512 (2.634–7.728)	< 0.001*
Benzodiazepines	9 (4.6%)	29 (8.9%)	0.491 (0.227–1.061)	0.070		
Non‐steroid anti‐inflammatory drugs	18 (9.2%)	26 (8.0%)	1.163 (0.620–2.181)	0.638		
Grade of gastroesophageal flap valve			1.590 (0.929–2.721)	0.091		
Grade I + II	167 (85.2%)	293 (90.2%)				
Grade III + IV	29 (14.8%)	32 (9.8%)				
Ectopic esophageal and astric mucosa	14 (7.1%)	15 (4.6%)	1.590 (0.750–3.369)	0.226		
Bile reflux	18 (9.2%)	26 (8.0%)	1.163 (0.620–2.181)	0.638		
Gastrointestinal ulcer	22 (11.2%)	13 (4.0%)	3.034 (1.491–6.174)	0.002*		
Mucosal atrophy/intestinalization	72 (36.7%)	115 (35.4%)	1.060 (0.733–1.533)	0.756		
Hp infection	68 (34.7%)	32 (9.8%)	4.864 (3.044–7.773)	< 0.001*	3.073 (1.692–5.581)	< 0.001*
GERD	125 (63.8%)	93 (28.6%)	4.392 (3.010–6.408)	< 0.001*	3.067 (1.919–4.902)	< 0.001*

*Note:* * indicated the statistically significant *p* < 0.05.

### 5.3 The Predictive Efficacy of Exposed Cardiac Glands on GERD

**FIGURE 2:**
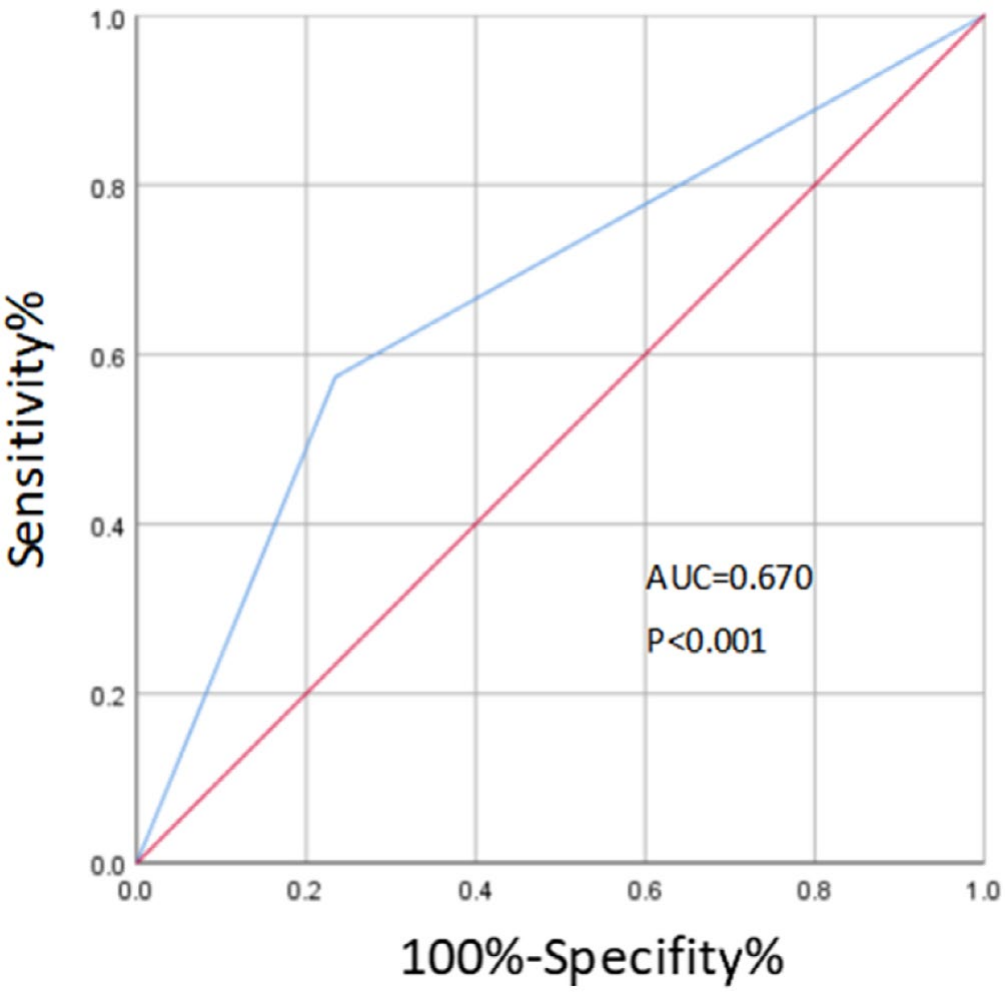
ROC curve of GERD predicted by exposed cardiac glands.

There were 125 cases (63.8%) of exposed cardiac glands with GERD, including 76 cases (60.8%) of NERD and 49 cases (39.2%) of RE. In conclusion, NERD appeared more frequently in patients with exposed cardiac glands with GERD. In patients with exposed cardiac glands with RE, exposed cardiac glands often appear around the damaged and eroded mucosa of the distal esophagus. ROC curve was established to evaluate the prediction effect of exposed cardiac gland on GERD, and the area under ROC curve (AUC) was 0.670 (*p* < 0.001), as shown in Figure 2.

### 5.4 Endoscopic Characteristics of the Exposed Cardiac Glands

#### 5.4.1 Basic Characteristics of Exposed Cardiac Glands

**FIGURE 3:**
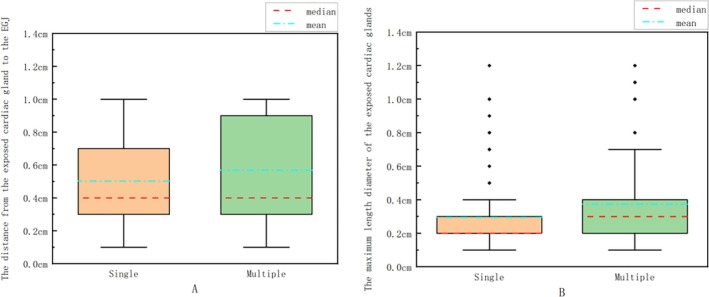
Box plot of the number of exposed cardiac glands and the distance from exposed cardiac glands to the EGJ (A); Box plot of the number of exposed cardiac glands and the maximum length diameter of exposed cardiac glands (B).

There were 119 cases (60.7%) with a single cardiac gland and 77 cases (39.3%) with multiple cardiac glands. The distance from the exposed cardiac gland to the EGJ was calculated (for patients with multiple exposed cardiac glands, the farthest exposed cardiac gland from the EGJ was selected); there were 132 cases (67.3%) with distance ≤ 0.5 cm and 64 cases (32.7%) with 0.5 cm < distance ≤ 1.0 cm. The maximum length diameter of the exposed cardiac glands was calculated, and there were 179 cases (91.3%) with length ≤ 0.5 cm, 17 cases (8.7%) with 0.5 cm < length ≤ 1.0 cm. Then, we drew box plots; the number of exposed cardiac glands was taken as the horizontal coordinate (single and multiple), and the distance from the exposed cardiac glands to the EGJ (for patients with multiple exposed cardiac glands, the farthest exposed cardiac gland from the EGJ was selected) and the maximum length diameter of exposed cardiac glands were taken as the ordinate coordinates, respectively, as shown in Figure 3. Here are the results: Median (P25, P75) values for the distance from the EGJ were calculated as follows: Single exposed cardiac glands: 0.4 (0.3, 0.7), Multiple exposed cardiac glands: 0.4 (0.3, 0.9). For maximum length, Single exposed cardiac glands: 0.2 (0.2, 0.3), Multiple exposed cardiac glands: 0.3 (0.2, 0.4). These results suggest that the majority of exposed cardiac glands were characterized by singularity, with a ≤ 0.5 cm distance from the EGJ and maximum length ≤ 0.5 cm, and patients with multiple ectopic cardiac glands exhibited significantly larger glandular lengths, as well as greater variation in both length dimensions and distances from the EGJ compared to single exposed cardiac glands.

#### 5.4.2 Exposed Cardiac Gland Circumferential Positioning

**FIGURE 4:**
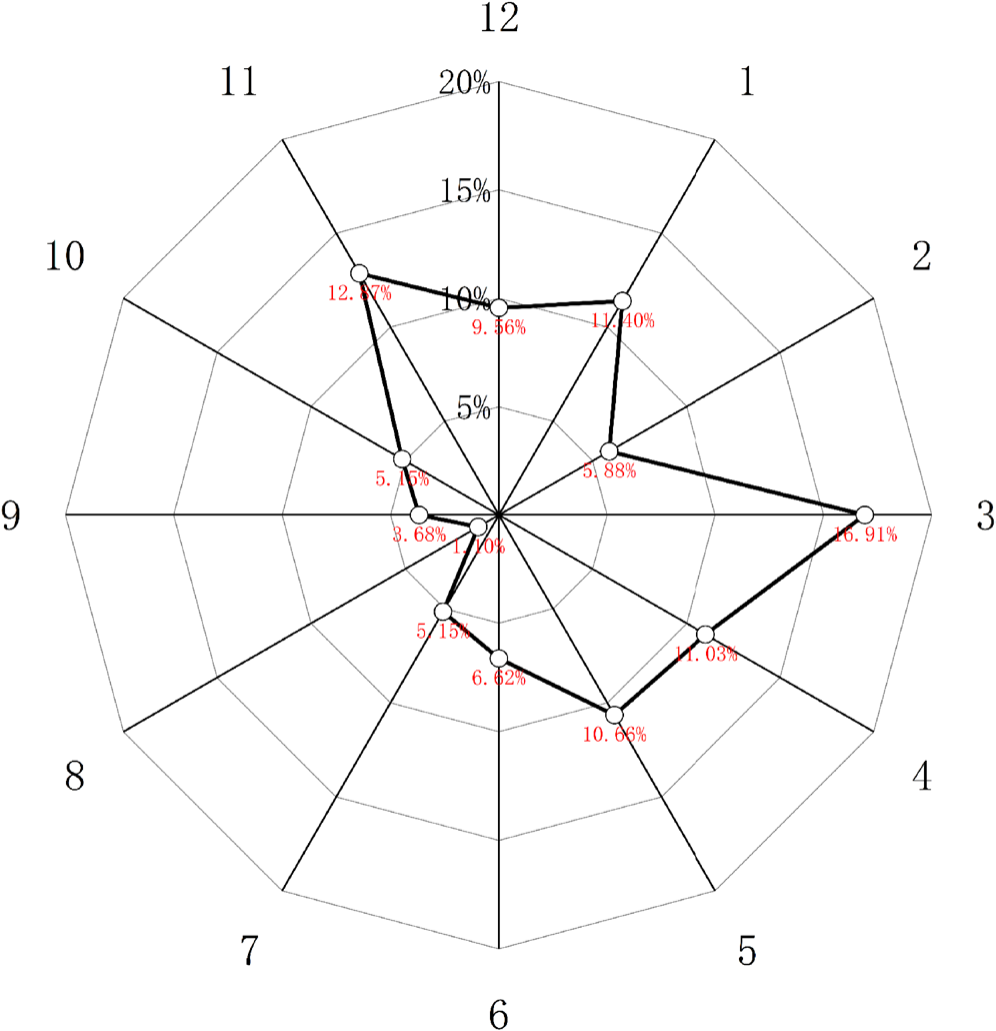
Circumferential distribution of exposed cardiac glands in the esophagus.

We set the direction of the lesser curvature of the esophagus as 12 o'clock of the clock, and the posterior wall as 3 o'clock of the clock, and drew a radar map according to the frequency of emergence of the exposed cardiac glands in different directions of the distal esophagus. Through the radar map, we found that the exposed cardiac glands tended to occur from 11 o'clock to 5 o'clock, that is, they appeared more frequently on the lesser curvature side and the posterior wall of the esophagus at the far end, which was basically consistent with the location of reflux. See Figure 4 for details.

#### 5.4.3 Typical Pictures of Exposed Cardiac Glands

**FIGURE 5:**
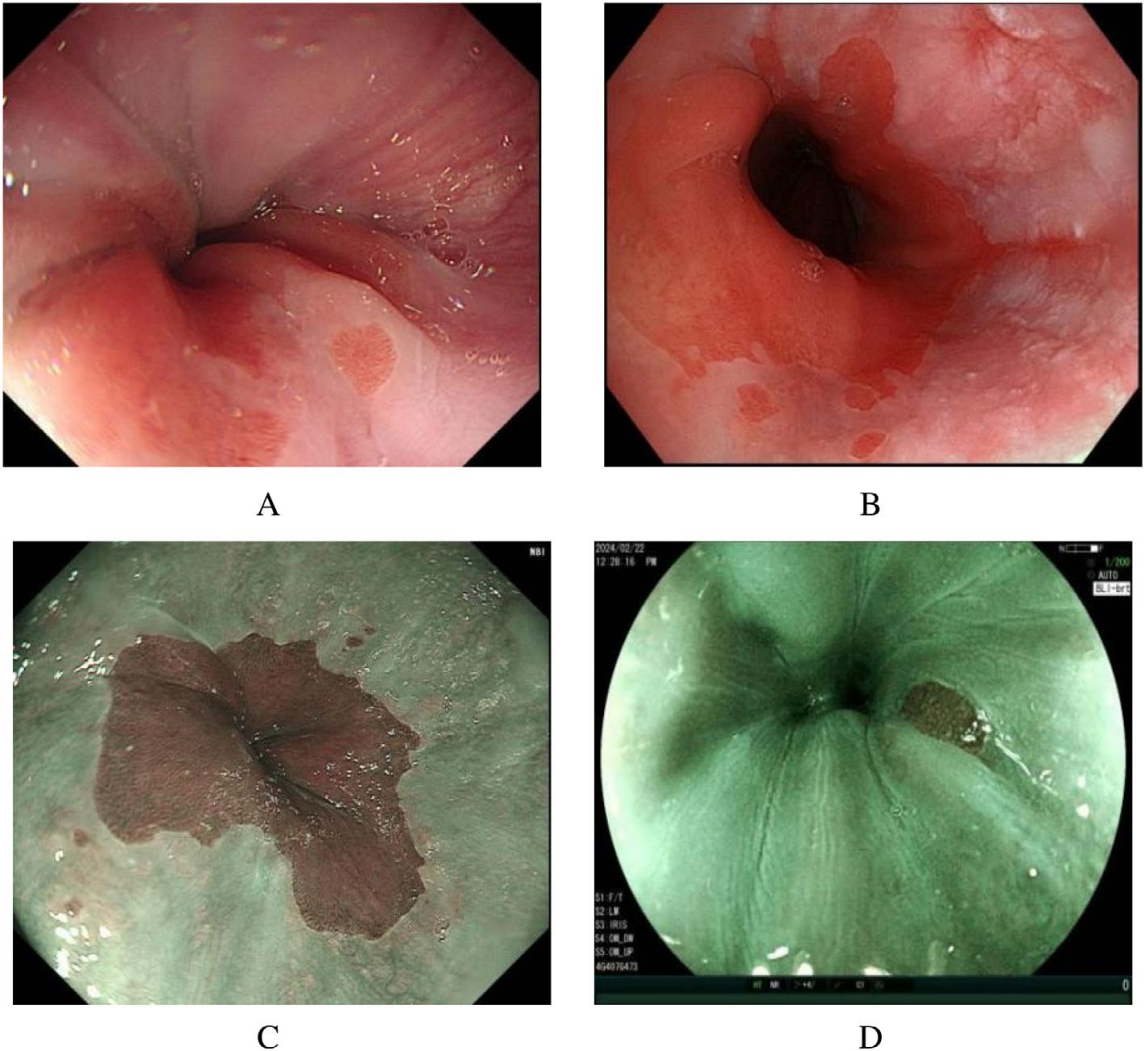
Typical pictures of exposed cardiac glands.

See Figure 5 for details.

## 6 Discussion

Statistical analysis in this study identified waist circumference, drinking, consumption of irritating foods, right side sleeping position, calcium antagonists, and Hp infection as significant risk factors for the development of exposed cardiac glands. Abdominal pressure will increase with the increase of waist circumference, and the pressure gradient of the diaphragm will become larger, resulting in the appearance or aggravation of GERD [9], and the EGJ will be continuously stimulated by reflux, which may lead to the appearance of exposed cardiac glands. Alcohol can directly damage the esophageal mucosa, reduce the esophageal motor function, and enhance the secretion of gastric juice [10]. In addition, alcohol can also cause hypersensitivity reactions in the esophagus, resulting in loose connections between esophageal epithelial cells, thus making it easier for reflux gastric acid to enter the esophageal epithelial cells [11]. The above factors jointly cause damage to the esophageal mucosa, which may lead to the exposure of the esophageal cardiac glands. Sadafi S et al. have suggested that eating irritating food can induce GERD and aggravate GERD symptoms [12]. Eating greasy food can increase gastric emptying time, stimulate gastric juice production, reduce esophageal sphincter pressure, and increase transient lower esophageal sphincter relaxation frequency, thus increasing the possibility of reflux [13, 14]. Repeated consumption of spicy food can reduce the efficiency of esophageal secondary peristalsis and prolong the acid clearance time of the esophagus. In addition, irritating food itself also has a certain stimulating effect on the esophagus, coupled with the friction effect of food clumps on the esophagus. The above factors work together to cause certain damage to the esophageal mucosa, which may lead to the occurrence of exposed cardiac glands. Due to different sleeping positions, the position of the esophagus and stomach will also change relatively to each other. In the right side sleeping position, the esophagus is lower than the stomach, which can induce more reflux, significantly prolonging the exposure time and clearance time of esophageal acid in patients. In addition, reflux caused by the right side sleeping position will be more serious when the lower esophageal sphincter is relaxed or the pressure is reduced [15], and reflux will continue to stimulate the distal esophagus. It may lead to chronic damage to the squamous epithelium of the distal esophagus, leading to the emergence of exposed cardiac glands. Calcium antagonists can reduce the lower esophageal sphincter pressure and impair esophageal clearance, thereby causing reflux or aggravating existing reflux symptoms [16]. Long‐term stimulation of reflux may induce the emergence of exposed cardiac glands. Hp can regulate the function of immune cells, avoid the killing effect of reactive oxygen species and active nitrogen produced by immune cells, and the uncontrolled immune response continues to produce the above two substances, which will cause mucosal damage, damage surrounding cells, and enhance inflammation [17]. In addition, Hp may also be fixed in the esophageal mucosa, aggravating esophageal inflammation [18], and a sustained inflammatory response and mucosal damage may lead to exposure of the esophageal cardiac glands. In terms of endoscopic factors, exposed cardiac glands were positively correlated with GERD, and we found that exposed cardiac glands were most common with single, shorter maximum length diameter and close distance to the EGJ. Compared with patients with a single exposed cardiac gland, the mean distances from exposed cardiac glands to the EGJ and the mean maximum length diameters of exposed cardiac glands were larger in patients with multiple exposed cardiac glands. Among the patients with exposed cardiac glands with GERD, NERD patients were more than RE patients. Yagi et al. [19] also drew a similar conclusion. They also said that esophageal cardiac glands play a protective role in the local esophageal squamous epithelium, which can avoid the damage of reflux. In our collection of patients with exposed cardiac glands with RE, exposed cardiac glands often appear in the periphery of mucosal damage and erosion. Therefore, based on the above information, we speculated that although esophageal cardiac glands have the function of protecting squamous epithelium, under the stimulation of reflux, the protective ability of some esophageal cardiac glands will gradually decline, resulting in the damage of the squamous epithelium. Because columnar epithelium is more tolerant to the acidic environment, this part of the esophageal cardiac glands will be exposed to the esophageal surface in order to adapt to the acidic environment caused by reflux. However, the rest of the esophageal cardiac gland still has a strong role in protecting the esophagus, so the lower esophageal mucosa will not appear obvious damage and erosion. With the continuous enhancement of reflux stimulation, the protective function of the esophageal cardiac glands decreases to a certain extent, which may lead to the emergence of RE, and the exposed cardiac glands may also change from fewer to more, from small to large, interwoven and fused to the proximal end of the esophagus, resulting in the emergence of Barrett's esophagus in severe cases. This study also provided a statistical analysis of the circumferential distribution of exposed cardiac glands in the distal esophagus. We positioned the mucosa of the lesser curvature of the esophagus at 12 o'clock and the esophageal posterior wall at 3 o'clock on the clock. According to the statistics, the exposed cardiac glands are most likely to occur at the distal end of the esophagus from 11 to 5 o'clock, roughly located in the lesser curvature side of the esophagus and the posterior wall of the esophagus. The results of Tomoko Katsube et al.'s study also showed that reflux tended to occur on the lesser curvature of the esophagus and the posterior wall of the esophagus, and the analysis may be related to the asymmetric pressure of the lower esophageal sphincter and supine sleep [20], and the two prone locations were roughly the same, basically verifying our above speculation.

Of course, this study has some limitations. First, our study was a single‐center study with a small sample size and lack of long‐term follow‐up. The second limitation is the heterogeneity of our population, especially in terms of age, which may affect the generalizability and extrapolation of our results. Our preliminary results need to be confirmed in future with large sample sizes, longer follow‐up times and multi‐center studies.

## 7 Conclusion

In summary, waist circumference, drinking, irritating food, right side sleeping position, calcium antagonists, Hp infection, and GERD are all independent risk factors for exposed cardiac glands, and exposed cardiac glands tend to occur on the lesser curvature and posterior wall of the lower esophagus, which is basically consistent with the sites prone to reflux. Affected by reflux, exposed cardiac glands may coalesce and progress. In addition, exposed cardiac glands have some predictive value for GERD. Clinically, for patients with exposed cardiac glands found in gastroscopy, in particular, patients with the above risk factors, we need to strengthen follow‐up and attach importance to them, actively preventing such disease.

## Ethics Statement

This study was approved by the Hospital Ethics Committee (approval number CDCHLL2024‐601). The procedures used in this study adhere to the tenets of the Declaration of Helsinki.

## Consent

Informed consent was obtained from all individual participants included in the study.

## Conflicts of Interest

The authors declare no conflicts of interest.

## Data Availability

The data that support the findings of this study are available from the corresponding author upon reasonable request.
